# Synergistic production of 20(*S*)-protopanaxadiol from protopanaxadiol-type ginsenosides by β-glycosidases from *Dictyoglomus turgidum* and *Caldicellulosiruptor bescii*

**DOI:** 10.1186/s13568-017-0524-9

**Published:** 2017-12-14

**Authors:** Ji-Hyeon Choi, Min-Ju Seo, Kyung-Chul Shin, Ki Won Lee, Deok-Kun Oh

**Affiliations:** 1 Department of Bioscience and Biotechnology, Konkuk University, Seoul, 05029 Republic of Korea; 2 BOBSNU Corporation R&D Center, Suwon, 16229 Republic of Korea; 3 Department of Agricultural Biotechnology, Seoul National University, Seoul, 08826 Republic of Korea

**Keywords:** α-l-Arabinopyranosidase activity, 20(*S*)-Protopanaxadiol, β-Glycosidase, *Dictyoglomus turgidum*, *Caldicellulosiruptor bescii*

## Abstract

20(*S*)-Protopanaxadiol (APPD) has potential uses in the pharmaceutical, cosmetic, and food industries because of its anti-stress, anti-fatigue, anti-cancer, anti-inflammatory, and anti-wrinkle properties. However, APPD production is difficult because β-glycosidases that convert the protopanaxadiol (PPD)-type ginsenoside compound K to APPD are rare. β-Glycosidase from *Dictyoglomus turgidum* (DT-bgl) has the highest specific activity for converting compound K to APPD, but exhibits no activity towards the α-l-arabinopyranoside moiety in compound Y. Therefore, β-glycosidase from *Caldicellulosiruptor bescii* (CB-bgl), which has a strong α-l-arabinopyranosidase activity, was used along with DT-bgl. The volumetric and specific productivities of the two-enzyme system for APPD using ginseng root extract were 38.4- and 38.7-fold higher, respectively, than those of β-glycosidase from *Pyrococcus furiosus*, which had the highest volumetric productivity previously reported, at the same enzyme and substrate concentrations. Thus, DT-bgl combined with CB-bgl completely converted PPD-type ginsenosides to APPD with the highest volumetric and specific productivities reported thus far.

**Electronic supplementary material:**

The online version of this article (10.1186/s13568-017-0524-9) contains supplementary material, which is available to authorized users.

## Introduction

Ginseng (*Panax ginseng* C. A. Meyer), which belongs to the *Araliaceae* family, is a slow-growing herb plant that has been used as a traditional medicine in Asia for centuries (Xiang et al. 2008). Ginseng has many beneficial effects, including improving mood and brain function, helping with weight loss, treating sexual dysfunction, inducing lower blood sugar levels, preventing cancer, and boosting the immune system (Gao et al. 2013b; Helms 2004; Jang et al. 2008; Kang and Min 2012; Reay et al. 2010; Xie et al. 2002). Therefore, ginseng has been widely utilized as an ingredient in functional foods and as a raw material in traditional medicine (Chung et al. 2011). Ginsenosides are the biologically and pharmacologically active components of ginseng. Most ginsenosides are divided into the protopanaxadiol (PPD)- and protopanaxatriol (PPT)-types and consist of a PPD or PPT aglycon and a sugar component containing 1–4 glycoside molecules such as glucose, arabinopyranose, arabinofuranose, xylose, and rhamnose. The glycosides are linked to a dammarane (tetracyclic triterpene) skeleton. Naturally occurring ginsenosides are mostly present as glycosylated forms, while their deglycosylated forms are present either in small amounts or not at all (Shin and Oh 2016). The deglycosylated forms of ginsenosides show greater biological and pharmacological activities than the glycosylated forms owing to their higher bioavailability and better absorption in the gastrointestinal tract (Kim et al. 2005). Therefore, to obtain biologically and pharmacologically active ginsenosides, specific sugar-hydrolysis techniques are required.

20(*S*)-Protopanaxadiol (aglycon protopanaxadiol, APPD) is a final metabolite of PPD-type ginsenosides that is not present in ginseng. The production of APPD is relatively difficult because it requires the complete hydrolysis of all the sugar moieties linked to the dammarane skeleton of PPD-type ginsenosides. APPD has been reported to have beneficial functional properties, including anti-stress, anti-fatigue, anti-cancer, anti-tumor, anti-inflammatory, and anti-wrinkle effects, and therefore it has potential uses in the pharmaceutical, cosmetic, and food industries (Chen et al. 2016; Gao et al. 2013a; Han et al. 2017; Oh et al. 2015). Although APPD is currently manufactured by chemical synthesis methods, those methods exhibit a low yield, selectivity, and productivity. In addition, they produce various side products, including the products of the cyclization, epimerization, hydroxylation, and hydration of the side chains, and contribute to environmental pollution. Alkaline hydrolysis has mostly been used for APPD production because it exhibits fewer undesired side reactions than acid hydrolysis does. However, alkaline hydrolysis does not exceed a yield of 80% and has a low productivity (Cui et al. 1993; Liu et al. 2010). In contrast, enzymatic transformation is highly selective for hydrolyzing the sugar moieties of ginsenosides without producing byproducts or causing environmental pollution.

Recently, β-glycosidases have been used to produce various minor ginsenosides with a high selectivity, yield, and productivity (Hong et al. 2012). β-Glycosidases from *Dictyoglomus turgidum* (Lee et al. 2012), *Pyrococcus furiosus* (Yoo et al. 2011), and *Terrabacter ginsenosidimutans* (Jin et al. 2012), as well as a glycoside oxidoreductase from *Rhizobium* sp. (Kim et al. 2012) have been used for APPD production. However, all those enzymes, except for the β-glycosidase from *D. turgidum* (DT-bgl), show a very low specific activity for the hydrolysis of the PPD-type ginsenoside compound K (C-K). Although DT-bgl shows the highest specific activity for APPD production, this enzyme has the critical drawback that it shows no α-l-arabinopyranosidase activity. Thus, DT-bgl cannot convert all PPD-type ginsenosides to APPD.

In this study, to convert all PPD-type ginsenosides to APPD with the highest productivity, β-glycosidase from *Caldicellulosiruptor bescii* (CB-bgl), which has a strong α-l-arabinopyranosidase activity, was used along with DT-bgl. The optimal reaction conditions of DT-bgl supplemented with CB-bgl were determined, including the pH, temperature, concentration ratio of CB-bgl to DT-bgl, and concentrations of substrate and enzymes. Under the optimal conditions, the two enzymes applied together completely converted all the PPD-type ginsenosides in ginseng root extract to APPD with the highest volumetric and specific productivities reported thus far.

## Materials and methods

### Materials

The ginsenoside standards compound Y (C-Y), C-K, and APPD were purchased from Ambo Institute (Seoul, Republic of Korea). Dried powder of *P. ginseng* root extract was purchased from Acegem (Jilin, China). All the solvents used in this experiment were purchased from Duksan (Ansan, Republic of Korea). Digoxin was purchased from Sigma-Aldrich (St. Louis. MO, USA), and it was used as an internal standard in a high-performance liquid chromatography (HPLC) analysis of ginsenosides.

### Gene cloning and culture conditions

β-Glycosidases from thermophilic bacteria including *D. turgidum* DSM 6724 (DSMZ, Braunschweig, Germany), *P. furiosus* DSM 3638, *Sulfolobus acidocaldarius* DSM 639, *Sulfolobus solfataricus* DSM 1617, and *Caldicellulosiruptor saccharolyticus* DSM 8903 were cloned as previously described, and these strains were used as the expression plasmids pET28a, pET24a, pTrc99a, pET24a, and pTrc99a, respectively (Lee et al. 2012; Noh and Oh 2009; Shin et al. 2013; Yoo et al. 2011). The gene of β-glucosidase from *C. bescii* DSM 6725 (Genbank accession number WP_015908678.1) was cloned by the one-step isothermal assembly method using pET24a vector (Gibson et al. 2009). *Escherichia coli* ER2566 expressing glycoside hydrolase was cultivated in a 2-l flask containing 450 ml of Luria–Bertani medium mixed with 20 μg ml^−1^ of ampicillin for pTrc99a vector and kanamycin for the other vectors at 37 °C with shaking at 200 rpm. When the optical density of the bacterial culture at 600 nm reached 0.6–0.8, 0.1 mM isopropyl-β-d-thiogalactopyranoside was added to the medium for inducing enzyme expression. The culture temperature and agitation were then reduced to 16 °C and 150 rpm, respectively, and the culture was incubated for a further 16 h.

### Enzyme preparation

*Escherichia coli* cells expressing glycoside hydrolase were harvested and suspended in citrate/phosphate buffer (pH 5.5) consisting of 50 mM Na_2_HPO_4_ and 50 mM citric acid. The suspended cells were lysed using a sonicator (Sonic Dismembrator Model 100; Fisher Scientific, Pittsburgh, PA, USA) on ice for 10 min. The unbroken cells and cell debris were eliminated by centrifugation at 13,000×*g* for 10 min at 4 °C, and the supernatant was heated at 70 °C for 10 min to remove proteins derived from *E. coli* and obtain the thermophilic target protein. After heat treatment, the suspension was centrifuged at 13,000×*g* for 10 min to eliminate insoluble aggregated proteins. The supernatant obtained was filtered using a 0.45-μm sterile syringe filter, and the filtrate was used as the purified enzyme. Expression of the purified enzyme was confirmed by sodium dodecyl sulfate polyacrylamide gel electrophoresis (SDS-PAGE).

### Specific activities of glycoside hydrolases towards C-Y and C-K

The specific activities of glycoside hydrolases from thermophilic bacteria towards ginsenosides C-Y and C-K were investigated. These two ginsenosides were used to determine the activities of the enzymes for hydrolyzing an α-l-arabinopyranoside in C-Y and a β-d-glucopyranoside in C-K, respectively. The reactions were performed in 50 mM citrate/phosphate buffer (pH 5.5) at 80–95 °C with enzyme concentrations ranging from 0.0005 to 0.4 mg ml^−1^ and reaction times ranging from 5 to 30 min. Only the part of each reaction that showed a linear correlation between product concentration and time was used for the determination of specific activity.

### Effects of substrate and enzyme concentrations on APPD production

The effect of the concentration of total PPD-type ginsenosides in ginseng root extract on APPD production was estimated by varying the concentration of PPD-type ginsenosides from 1.1 to 5.4 mM in the extract while maintaining a fixed DT-bgl concentration of 2 mg ml^−1^ for 4 h. The effect of DT-bgl concentration on APPD production was investigated by varying the enzyme concentration from 0.5 to 12 mg ml^−1^ while maintaining a fixed concentration of 2.8 mM PPD-type ginsenosides. In these experiments, the reaction times were reduced to 1 h to discern distinct differences at high concentrations of enzyme. Under the optimized conditions, the time-course reactions for the conversion of PPD-type ginsenosides in ginseng root extract to APPD were performed at 80 °C in 50 mM citrate/phosphate buffer (pH 5.5) containing 2.8 mM PPD-type ginsenosides and 8 mg ml^−1^ DT-bgl. The effect of the CB-bgl concentration on the production of APPD from C-Y as remaining substrate was investigated by varying the enzyme concentration from 0.0025 to 0.1 mg ml^−1^ while maintaining a fixed concentration of 8 mg ml^−1^ DT-bgl for 2 h.

### Production of APPD from PPD-type ginsenosides in ginseng root extract

The production of APPD was performed in 50 mM citrate/phosphate buffer (pH 5.5) containing 2.8 mM PPD-type ginsenosides in ginseng root extract, 8 mg ml^−1^ DT-bgl supplemented with 0.05 mg ml^−1^ CB-bgl or 8.05 mg ml^−1^ PF-bgl at 80 or 95 °C, respectively.

### Analytical methods

All the reactions were stopped and extracted by adding an equal volume of *n*-butanol with digoxin as an internal standard. The solvent in the extracted solution was then removed using an evaporator, and methanol was added to the dried sample. Ginsenosides were analyzed by an HPLC system (Agilent 1100, Agilent, Santa Clara, CA, USA) equipped with an ultraviolet–visible light detector at a wavelength of 203 nm and a C_18_ column (YMC, Kyoto, Japan). The column was eluted at 37 °C with a linear gradient of acetonitrile and water from 30:70 to 90:10 (v/v) for 80 min at a flow rate of 1 ml min^−1^. The ginsenosides in the reaction samples obtained were identified as the same retention times of the ginsenoside standards. The amounts of ginsenosides were determined using linear calibration curves, relating the peak areas to the concentrations of ginsenoside standards.

## Results

### Contents of PPD- and PPT-type ginsenosides in ginseng extract powder

The composition of major ginsenosides in the dried powder of the ginseng root extract used in this study was analyzed by HPLC (Additional file 1: Table S1). The PPD-type major ginsenosides comprised 86.2% (w/w) of the total major ginsenosides. Their contents followed the decreasing order Rb_1_ (26.9% of total major ginsenosides), Rc (25.7%), Rb_2_ (17.1%), and Rd (16.5%), while the contents of PPT-type major ginsenosides followed the decreasing order Re (10.2%), Rg_1_ (2.5%), Rg_2_ (0.6%), and F_1_ (0.4%).

### Selection of a main enzyme for APPD production

PF-bgl shows hydrolytic activities towards glucose, arabinopyranose, and arabinofuranose linked to the dammarane skeleton of PPD-type ginsenosides and has been reported to completely convert all PPD-type ginsenosides to APPD with the highest APPD productivity among the previous methods (Yoo et al. 2011). DT-bgl has been also reported to convert C-K to APPD. PF-bgl and CB-bgl were purified by heating at 70 °C for 10 min, and the purified enzymes showed a single band in SDS-PAGE (Additional file 1: Figure S1). In order to evaluate the APPD-producing activity of PF-bgl and DT-bgl, the specific activities of these two enzymes for the production of APPD from C-K were determined (Table 1). The specific activity of DT-bgl was 7.6-fold higher than that of PF-bgl. Enzymes that convert compound K to APPD by hydrolyzing glucose linked to C-20 in compound K are extremely rare. Although PF-bgl has the highest productivity among the few enzymes, DT-bgl shows significantly higher specific activity than PF-bgl, indicating that DT-bgl is the most efficient APPD producer. Therefore, DT-bgl was selected as the main enzyme for APPD production in the present study.

**Table 1:** Specific activity of glycoside hydrolases from thermophilic bacteria towards ginsenosides C-Y and C-K

Microorganism	Enzyme	Specific activity (nmol min^−1^ mg^−1^)	
		Substrate (product)	
		C-Y (C-K)	C-K (APPD)
*Dictyoglomus turgidum*	β-Glycosidase	0	16.7 ± 0.3
*Pyrococcus furiosus*	β-Glycosidase	1750 ± 20	2.2 ± 0.1
*Caldicellulosiruptor saccharolyticus*	β-Galactosidase	3510 ± 30	0
*Caldicellulosiruptor bescii*	β-Glycosidase	37,900 ± 110	0
*Sulfolobus acidocaldarius*	β-Glycosidase	1.0 ± 0.1	0
*Sulfolobus solfataricus*	β-Glycosidase	2200 ± 30	0

### Optimization of the reaction conditions of DT-bgl for APPD production

**Fig. 1:**
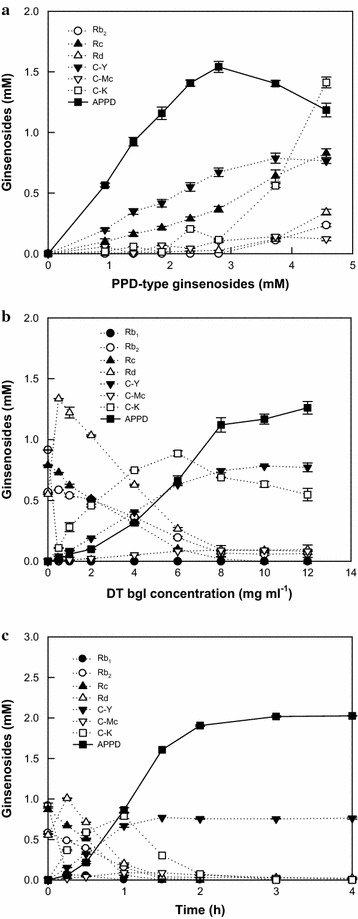
Optimization of **a** the concentration of total protopanaxadiol (PPD)-type ginsenosides in ginseng root extract as substrates; **b** the concentration of the β-glycosidase from *Dictyoglomus turgidum* (DT-bgl) as an enzyme for 20(*S*)-protopanaxadiol (APPD) production; and **c** the time-course reactions for the production of APPD from PPD-type ginsenosides in ginseng root extract by DT-bgl alone under the optimal conditions. Data represent the means of three experiments and error bars represent the standard deviation

For the increased production of APPD by DT-bgl, the optimal reaction conditions were determined. The activity of DT-bgl for ginsenoside Rd was maximal at pH 5.5 and 80 °C, and its half-life at that temperature was 11 h (Lee et al. 2012). The reaction was completed within 4 h, indicating that the enzyme is stable at 80 °C during the reaction. Thus, the optimal pH and temperature for the production of APPD from PPD-type ginsenosides were selected as pH 5.5 and 80 °C, respectively. For the determination of the optimal concentration of total major PPD-type ginsenosides (Rb_1_, Rb_2_, Rc, and Rd) in ginseng root extract for APPD production, the reactions were performed with 2 mg ml^−1^ DT-bgl at various concentrations of the PPD-type ginsenosides between 0.93 and 4.67 mM for 4 h (Fig. 1a). The maximal production of APPD was observed with 2.8 mM PPD-type ginsenosides in ginseng root extract. Thus, the optimal concentration of total major PPD-type ginsenosides was 2.8 mM. The effect of DT-bgl concentration on APPD production was investigated with 2.8 mM PPD-type ginsenosides in ginseng root extract by varying the concentration of DT-bgl between 0.5 and 12 mg ml^−1^ for 1 h. To distinctly demonstrate the effect of enzyme concentration on APPD production at the higher concentrations of the enzyme, the reaction time was reduced to 1 h. APPD production from ginseng root extract increased when the enzyme concentration was increased; however, the amount of additional APPD production per mg of added enzyme significantly decreased above 8 mg ml^−1^ DT-bgl (Fig. 1b), indicating that the optimal enzyme concentration was 8 mg ml^−1^.

### APPD production by DT-bgl under the optimal conditions

The optimal reaction conditions for APPD production were pH 5.5, 80 °C, 2.8 mM PPD-type ginsenosides in ginseng root extract, and 8 mg ml^−1^ DT-bgl. Under the optimal conditions, the biotransformation of PPD-type ginsenosides in ginseng root extract to APPD by DT-bgl was performed for 4 h (Fig. 1c). The HPLC profiles obtained during the reaction are presented in Additional file 1: Figure S2. After 3 h, all PPD-type ginsenosides except for C-Y were converted to APPD. C-Y was not hydrolyzed and thus its concentration remained stable throughout the reaction because DT-bgl exhibited no α-l-arabinopyranosidase activity. The enzyme produced 2 mM APPD for 3 h, with a volumetric productivity of 667 µM h^−1^, a specific productivity of 83 µmol g^−1^ h^−1^, and a molar conversion of 71%.

### Selection of the additional enzyme for increasing the production of APPD by hydrolyzing C-Y

To increase APPD productivity for total PPD-type ginsenosides in ginseng extract, the specific activities of several β-glycosidases derived from thermophilic bacteria known to hydrolyze α-l-arabinopyranoside in C-Y were measured (Table 1). β-Glycosidases from thermophilic bacteria were purified by heating at 70 °C for 10 min, and the purified enzymes showed a single band in SDS-PAGE (Additional file 1: Figure S1). Among the tested β-glycosidases, CB-bgl showed the highest activity towards C-Y and its activity was 10.8-fold higher than that of the β-glycosidase from *C. saccharolyticus*, which exhibited the second highest activity. Thus, CB-bgl was selected as the additional enzyme to compensate for the lack of α-l-arabinosidase activity by DT-bgl, and the combination of DT-bgl and CB-bgl was expected to increase the productivity of APPD by converting the accumulated C-Y into APPD.

### Determination of the optimal CB-bgl concentration for the complete hydrolysis of α-l-arabinopyranoside in C-Y

**Fig. 2:**
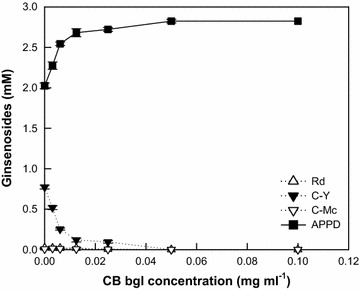
Determination of the optimal concentration of CB-bgl for the complete conversion of the remaining PPD-type ginsenosides in the reaction solution after a 3-h reaction with DT-bgl for the biotransformation of 2.8 mM PPD-type ginsenosides in ginseng root extract to APPD. Data represent the means of three experiments and error bars represent the standard deviation

After the biotransformation of 2.8 mM PPD-type ginsenosides in ginseng root extract to APPD by DT-bgl for 3 h, the reaction solution contained DT-bgl, APPD, and ginsenoside C-Y without other PPD-type ginsenosides. CB-bgl at concentrations ranging from 0.0025 to 0.1 mg ml^−1^ was added to the reaction solution with 8 mg ml^−1^ DT-bgl, and the mixture was incubated for another 2 h (Fig. 2). At concentrations above 0.05 mg ml^−1^ CB-bgl, the remaining C-Y was completely converted to APPD by the added CB-bgl. Therefore, the optimal concentration of CB-bgl for APPD production is 0.05 mg ml^−1^.

### APPD production from PPD-type ginsenosides in ginseng root extract by DT-bgl supplemented with CB-bgl under the optimal conditions

**Fig. 3:**
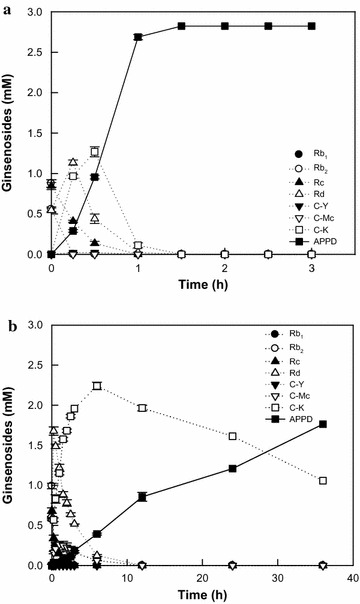
Time-course reaction for the production of APPD from PPD-type ginsenosides in ginseng root extract by **a** DT-bgl supplemented with CB-bgl and **b** PF-bgl. Data represent the means of three experiments and error bars represent the standard deviation

The optimal reaction conditions for APPD production were pH 5.5, 80 °C, 2.8 mM PPD-type ginsenosides in ginseng root extract, 8 mg ml^−1^ DT-bgl, and 0.05 mg ml^−1^ CB-bgl. Under the optimal conditions, time-course reactions for the production of APPD from PPD-type ginsenosides in ginseng root extract were performed (Fig. 3a). APPD production was compared with that obtained using PF-bgl alone, which had the highest volumetric productivity previously reported, at the same concentrations of total enzyme(s) (8.05 mg ml^−1^) and substrate (2.8 mM PPD-type ginsenosides) (Fig. 3b). The HPLC profiles obtained during the reactions are presented in Additional file 1: Figure S3. DT-bgl supplemented with CB-bgl and PF-bgl converted 2.8 mM PPD-type ginsenosides in ginseng root extract to 2.8 and 1.8 mM APPD for 1.5 and 36 h, respectively, with volumetric productivities of 1880 and 49 µM h^−1^, specific productivities of 235 and 6.1 µmol g^−1^ h^−1^, and molar conversions of 100 and 61%, respectively.

## Discussion

The composition of PPD- and PPT-type major ginsenosides in ginseng root extract followed the order Rb_1_ (26.9%) > Rc (25.7%) > Rb_2_ (17.1%) > Rd (16.5%), and Re (10.2%) > Rg_1_ (2.5%) > Rg_2_ (0.6%) > F_1_ (0.4%), respectively (Additional file 1: Table S1). The content of ginsenoside Rd, which was the least abundant of the PPD-type major ginsenosides, was about 1.6-fold higher than that of ginsenoside Re, which was the most abundant of the PPT-type major ginsenosides. The content of total major PPD-type ginsenosides was 6.2-fold higher than that of total major PPT-type ginsenosides. The concentration ratios of PPD-type to PPT-type ginsenosides for Korean red ginseng, American ginseng (*Panax quinquefolius*) root and seed, *P. ginseng* root, and *Panax notoginseng* root were reported as 1.6, 1 and 2.7, 0.8, and 0.7, respectively (Ko et al. 2008; Lee et al. 2014; Schlag and McIntosh 2006; Shin et al. 2015). Commercial ginseng extract powder is the best substrate for APPD production based on the utilization efficiency of ginsenosides because the ratio of PPD-type to PPT-type ginsenosides in the powder form is the highest as 6.2 among the different types of ginseng extracts.

**Fig. 4:**
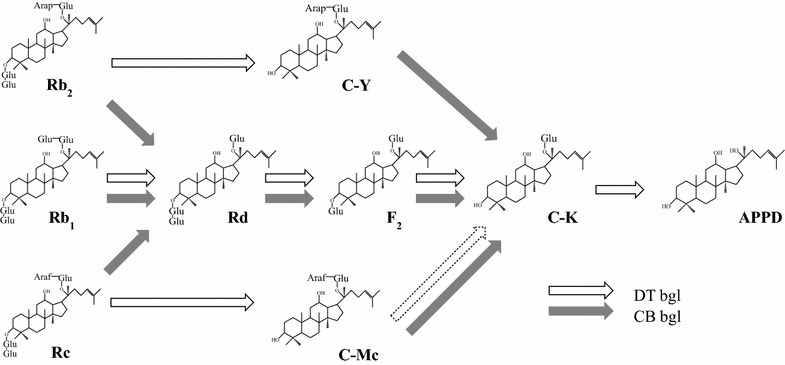
Biotransformation pathway for the conversion of ginsenosides Rb_1_, Rb_2_, and Rc to APPD via Rd, F_2_, C-K, C-Y, and C-Mc by DT-bgl supplemented with CB-bgl. The dotted line indicates a weak activity

The activity of DT-bgl for the conversion of Rb_1_ to APPD was the highest among the reported enzymatic and whole-cell methods. Therefore, DT-bgl was selected as the main enzyme for APPD production. DT-bgl catalyzes the reaction pathway of Rb_1_ → Rd → F_2_ → C-K → APPD. This enzyme also catalyzes the two reaction pathways of Rb_2_ → C-Y and Rc → compound Mc (C-Mc) → C-K because it does not hydrolyze α-l-arabinopyranoside in Rb_2_ and C-Y or α-l-arabinofuranoside in Rc (Lee et al. 2012). When a high concentration of enzyme was used, DT-bgl hydrolyzed α-l-arabinofuranose in C-Mc, but not α-l-arabinopyranose in C-Y. For the effective production of APPD, CB-bgl was selected as the second enzyme because it showed the highest activity towards C-Y (Table 1). CB-bgl has two transformation pathways of Rb_1_, Rb_2_, or Rc → Rd → F_2_ → C-K and C-Y or C-Mc → C-K (Fig. 4). Thus, CB-bgl hydrolyzed the α-l-arabinofuranoside and α-l-arabinopyranoside in C-Mc and C-Y to produce C-K, respectively, which were both transformed to APPD by DT-bgl. Although CB-bgl did not independently produce APPD, DT-bgl applied together with CB-bgl converted all the PPD-type ginsenosides in ginseng root extract to APPD via three transformation pathways, namely Rb_1_ → Rd → F_2_ → C-K → APPD, Rb_2_ → C-Y → C-K → APPD, and Rc → C-Mc → C-K → APPD.

When CB-bgl, which contained high α-l-arabinopyranosidase activity, at a 160-fold lower concentration than that of DT-bgl was added to DT-bgl, the remaining C-Y was completely decomposed. As a result, DT-bgl supplemented with CB-bgl resulted in the 100% conversion of all PPD-type ginsenosides in ginseng extract to APPD (Fig. 2). Thus, CB-bgl is an effective second enzyme for increasing APPD productivity. The time-course reactions of DT-bgl supplemented with CB-bgl were compared with those of PF-bgl (Fig. 3), which showed the previously highest APPD productivity (Yoo et al. 2011). DT-bgl supplemented with CB-bgl converted all PPD-type ginsenosides in ginseng extract to APPD for 1.5 h. However, PF-bgl showed 61% molar conversion to APPD for 36 h because PF-bgl had a relatively low hydrolyzing activity for glucose at C-20 in C-K. The volumetric productivity, specific productivity, and molar conversion obtained using DT-bgl supplemented with CB-bgl were 38.4-, 38.7-, and 1.6-fold higher than those obtained using PF-bgl alone and 2.8-, 2.8-, and 1.4-fold higher than those obtained using DT-bgl alone, respectively. Therefore, DT-bgl supplemented with CB-bgl is an efficient biocatalysis system for the production of APPD from PPD-type ginsenosides and contributes to an increased productivity of APPD.

The quantitative production of APPD from PPD-type ginsenosides and different types of ginseng extracts as substrates by microbial enzymes is presented in Table 2. DT-bgl converted 0.5 mM Rb_1_ to 0.2 mM APPD with a specific productivity of 36.4 µmol g^−1^ h^−1^ (Lee et al. 2012), which was the highest specific productivity previously reported. The specific productivity of APPD from ginseng root extract obtained using DT-bgl supplemented with CB-bgl was 6.4-fold higher than that obtained from Rb_1_ using DT-bgl alone. PF-bgl converted 4 mM Rd to 4 mM PPD-type ginsenosides in ginseng extract with a volumetric productivity of 667 µM h^−1^ and a specific productivity of 3.7 µmol g^−1^ h^−1^ (Yoo et al. 2011), which was the highest volumetric productivity previously reported. The volumetric and specific productivities of DT-bgl supplemented with CB-bgl were 2.8- and 63.5-fold higher than those of PF-bgl alone, respectively. Thus, DT-bgl supplemented with CB-bgl completely converted all PPD-type ginsenosides in ginseng root extract to APPD with the highest volumetric and specific productivities reported to date.

**Table 2:** APPD production from PPD-type ginsenosides and ginseng root extract by microbial enzymes

Microorganism	Enzyme	Substrate (mM)	APPD (mM)	Molar conversion (%)	Volumetric productivity (µM h^−1^)	Specific productivity (µmol g^−1^ h^−1^)	References
*Dictyoglomus turgidum*	β-Glycosidase	Rb_1_ (0.5)	0.2	40	33	36.4^a^	Lee et al. (2012)
*Pyrococcus furiosus*	β-Glycosidase	Rd (4.0)	4.0	100	800	8.9^a^	Yoo et al. (2011)
*Aspergillus niger*	Crude enzyme	Rg_3_ (0.6)	0.6	100	20	> 0.1	Liu et al. (2010)
*Pyrococcus furiosus*	β-Glycosidase	Ginseng root extract (4.0)	4.0	100	667	3.7^a^	Yoo et al. (2011)
*Pyrococcus furiosus*	β-Glycosidase	Ginseng root extract (2.8)	1.8	61	49	6.1	This study
*Dictyoglomus turgidum*	β-Glycosidase	Ginseng root extract (2.8)	1.9	71	667	83	This study
*Dictyoglomus turgidum* and *Caldicellulosiruptor bescii*	β-Glycosidase and β-glycosidase	Ginseng root extract (2.8)	2.8	100	1880	235	This study

The numbers in parentheses next to the substrates represent the substrate concentration

^a^Represents the calculated values based on the data in the corresponding reference

In conclusion, DT-bgl is an efficient APPD producer because it has a higher hydrolytic activity towards the β-d-glucopyranoside of C-K than that of PF-bgl, which had the highest volumetric productivity previously reported. However, DT-bgl has the critical drawback that it lacks any α-l-arabinopyranosidase activity, meaning that it cannot hydrolyze C-Y. To solve this problem, CB-bgl, which showed the highest activity for C-Y hydrolysis among several thermophilic glycoside hydrolases, was applied alongside DT-bgl. DT-bgl supplemented with CB-bgl completely converted all the PPD-type ginsenosides from ginseng extract to APPD. To the best of our knowledge, this combined enzyme system has the highest volumetric and specific productivities for APPD production reported so far.

## Additional file

**Additional file 1: Figure S1.** SDS-PAGE analysis of purified enzyme. Lane 1 *marker* proteins; lane 2 DT-bgl (85 kDa); lane 3 β-glycosidase from *P. furiosus* (55 kDa); lane 4 β-galactosidase from *C. saccharolyticus* (79 kDa); lane 5 CB-bgl (53 kDa); lane 6 β-glycosidase from *S. acidocaldarius* (57 kDa); and lane 7 β-glycosidase from *S. solfataricus* (57 kDa). **Figure S2.** HPLC profiles obtained during the conversion of PPD-type ginsenosides in ginseng root extract to APPD by DT-bgl alone. **Figure S3.** HPLC profiles obtained during the conversion of PPD-type ginsenosides in ginseng root extract to APPD by **a** DT-bgl supplemented with DT-bgl and **b** *P. furiosus* β-glycosidase. **Table S1.** Contents of protopanaxadiol (PPD)- and protopanaxatriol (PPT)-type ginsenosides in ginseng root extract powder.

## Abbreviations

APPD: 20(*S*)-protopanaxadiol
PPD: protopanaxadiol
DT-bgl: β-glycosidase from *Dictyoglomus turgidum*
CB-bgl: β-glycosidase from *Caldicellulosiruptor bescii*
PPT: protopanaxatriol
C-K: compound K
C-Y: compound Y
HPLC: high-performance liquid chromatography
SDS-PAGE: sodium dodecyl sulfate polyacrylamide gel electrophoresis
