# Supplementary material for: Synergistic production of 20(S)-protopanaxadiol from protopanaxadiol-type ginsenosides by β-glycosidases from Dictyoglomus turgidum and Caldicellulosiruptor bescii
Source: AMB Express. 2017 Dec 14;7:219. doi: 10.1186/s13568-017-0524-9 (PMC5730542; doi:10.1186/s13568-017-0524-9)
Supplement: Supplementary file 1 — Additional file 1: Figure S1. SDS-PAGE analysis of purified enzyme. Lane 1 marker proteins; lane 2 DT-bgl (85 kDa); lane 3 β-glycosidase from P. furiosus (55 kDa); lane 4 β-galactosidase from C. saccharolyticus (79 kDa); lane 5 CB-bgl (53 kDa); lane 6 β-glycosidase from S. acidocaldarius (57 kDa); and lane 7 β-glycosidase from S. solfataricus (57 kDa). Figure S2. HPLC profiles obtained during the conversion of PPD-type ginsenosides in ginseng root extract to APPD by DT-bgl alone. Figure S3. HPLC profiles obtained during the conversion of PPD-type ginsenosides in ginseng root extract to APPD by a DT-bgl supplemented with DT-bgl and b P. furiosus β-glycosidase. Table S1. Contents of protopanaxadiol (PPD)- and protopanaxatriol (PPT)-type ginsenosides in ginseng root extract powder. [file 13568_2017_524_MOESM1_ESM.docx]

**Additional data**

**AMB Express**

Synergistic production of 20(*S*)-protopanaxadiol from protopanaxadiol-type ginsenosides by β-glycosidases from *Dictyoglomus turgidum* and *Caldicellulosiruptor bescii*

Ji-Hyeon Choi, Min-Ju Seo, Kyung-Chul Shin, Ki Won Lee, Deok-Kun Oh

Corresponding author: D.-K. Oh

Department of Bioscience and Biotechnology, Konkuk University, Seoul 05029, Republic of Korea, E-mail: [deokkun@konkuk.ac.kr](mailto:deokkun@konkuk.ac.kr), Tel.: +82-2-454-3118; Fax: +82-2-444-5518


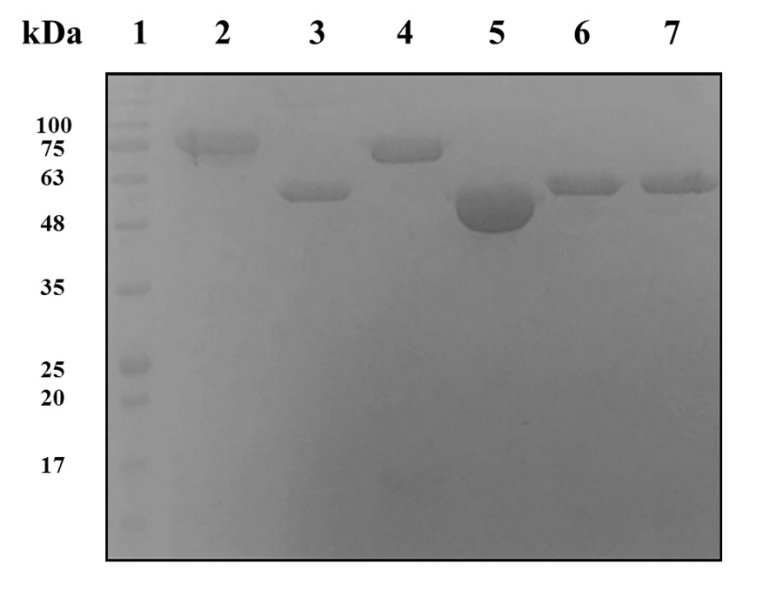


**Fig. S1** SDS-PAGE analysis of purified enzyme. *Lane 1* marker proteins; *lane 2* DT-bgl (85 kDa); *lane 3* β-glycosidase from *P. furiosus* (55 kDa); *lane 4* β-galactosidase from *C. saccharolyticus* (79 kDa); *lane 5* CB-bgl (53 kDa); *lane 6* β-glycosidase from *S. acidocaldarius* (57 kDa); and *lane 7* β-glycosidase from *S. solfataricus* (57 kDa)*.*

**
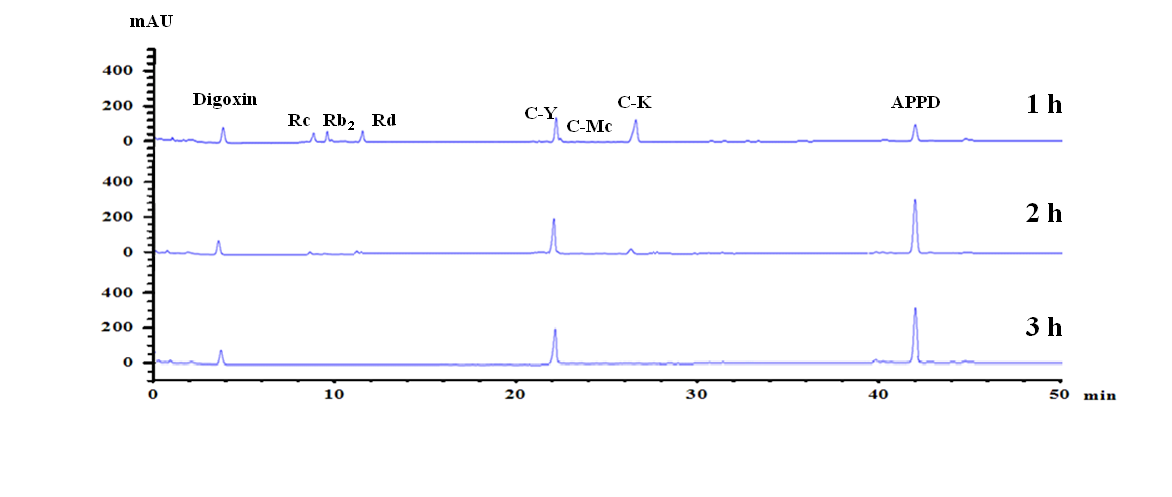
**

**Fig. S2** HPLC profiles obtained during the conversion of PPD-type ginsenosides in ginseng root extract to APPD by DT-bgl alone.

**a**


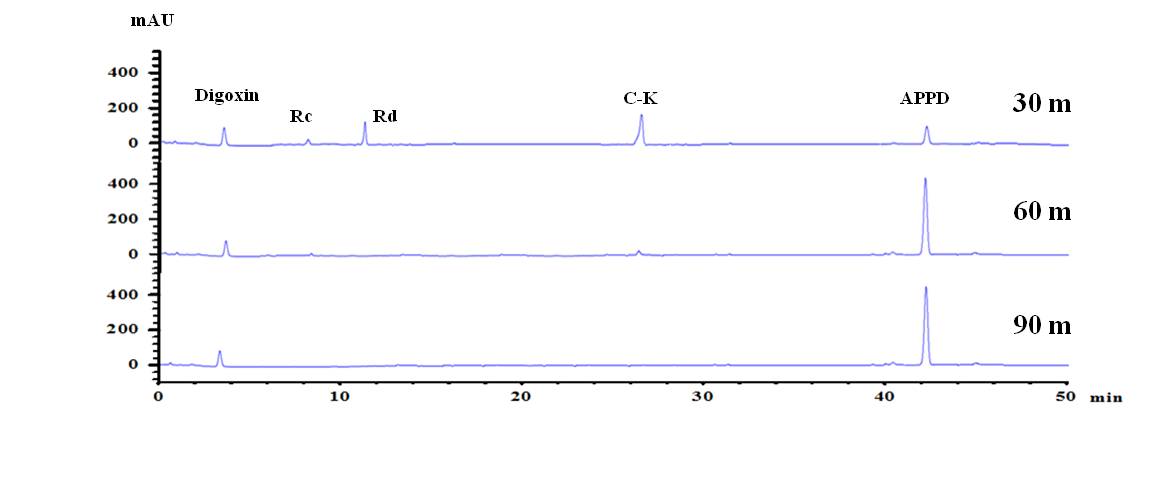


**b**

**
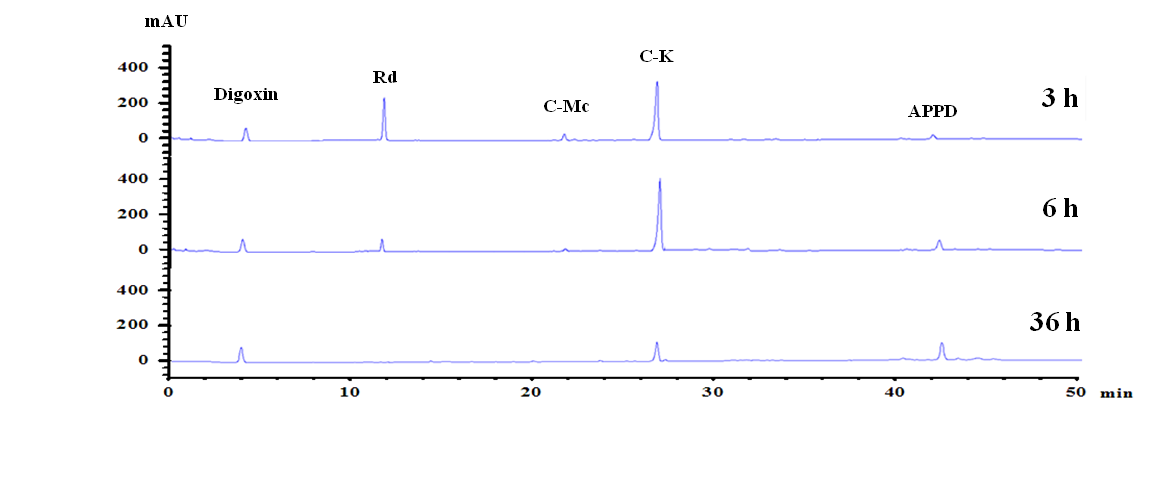
**

**Fig. S3** HPLC profiles obtained during the conversion of PPD-type ginsenosides in ginseng root extract to APPD by **a** DT-bgl supplemented with DT-bgl and **b** *P. furiosus* β-glycosidase.

**Table S1**

Contents of protopanaxadiol (PPD)- and protopanaxatriol (PPT)-type ginsenosides in ginseng root extract powder.

| Ginsenoside | Ginseng root extract | |
| --- | --- | --- |
|  | Concentration ratio (mg g^−1^) | Content (%, w/w) |
| PPD-type | | |
| Rb_1_ | 40.8 | 26.9 |
| Rb_2_ | 25.9 | 17.1 |
| Rc | 39.0 | 25.7 |
| Rd | 25.0 | 16.5 |
| Subtotal | 130.7 | 86.2 |
| PPT-type | | |
| Re | 15.5 | 10.2 |
| Rg_1_ | 3.8 | 2.5 |
| Rg_2_ | 1.0 | 0.6 |
| F_1_ | 0.7 | 0.4 |
| Subtotal | 20.9 | 13.8 |
| Total | 151.6 | 100.0 |
| PPD/PPT | 6.2 | |
